# Supplementary material for: Covariation of viral recombination with single nucleotide variants during virus evolution revealed by CoVaMa
Source: Nucleic Acids Res. 2022 Jan 8;50(7):e41. doi: 10.1093/nar/gkab1259 (PMC9023271; doi:10.1093/nar/gkab1259)

Supplementary Materials.

Covariation of viral recombination with single nucleotide variants during virus evolution revealed by CoVaMa

Shiyi Wang[1,2,3], Stephanea L. Sotcheff[4], Christian M. Gallardo[1,2], Elizabeth Jaworski[4], Bruce E. Torbett*[1,2,3,5] and Andrew L. Routh*[4,6,7]

1) Department of Immunology and Microbiology, The Scripps Research Institute, La Jolla, CA, USA
2) Center for Immunity and Immunotherapies, Seattle Children's Research Institute, Seattle, WA, USA
3) Department of Laboratory Medicine and Pathology, University of Washington, Seattle, WA, USA
4) Department of Biochemistry and Molecular Biology, University of Texas Medical Branch, Galveston, TX, USA
5) Department of Pediatrics, University of Washington School of Medicine, Seattle, WA, USA
6) Institute for Human Infections and Immunity, University of Texas Medical Branch, Galveston, TX, USA
7) Sealy Center for Structural Biology and Molecular Biophysics, University of Texas Medical Branch, Galveston, TX, USA.

Supplementary Table 1

Supplementary Table 1. The 15 associations with the highest wLD values detected in FHV RNA2 in Passage 9. (3sigma = 0.002992)

ID	NT1	NT2	wLD	wR2	wLDmax	correlation	Contingency table
1	575	736_to_1219	0.160452	0.649882	0.189137	('A', 'Pos', 'G', 'Neg')	[58, 478, 6, 7, 1364, 93, 7, 9]
2	575	250_to_513	0.136847	0.648261	0.167558	('A', 'Pos', 'G', 'Neg')	[57, 342, 6, 7, 1367, 61, 7, 9]
3	575	248_to_512	0.13225	0.593341	0.162764	('A', 'Pos', 'G', 'Neg')	[57, 338, 6, 2, 1367, 88, 7, 3]
4	1152	1149_to_1152	0.129885	0.691951	0.134186	('T', 'Pos', 'C', 'Neg')	[6, 0, 23, 78, 0, 0, 356, 2]
5	248_to_512	736_to_1219	0.106939	0.818994	0.123493	Rec-Rec	[1389, 27, 10, 205]
6	250_to_513	736_to_1219	0.106139	0.761762	0.122523	Rec-Rec	[1389, 27, 24, 208]
7	226	736_to_1219	0.099852	0.204686	0.110301	('A', 'Pos', 'G', 'Neg')	[640, 582, 12, 2, 640, 20, 16, 2]
8	226	248_to_512	0.092589	0.190669	0.099929	('A', 'Pos', 'G', 'Neg')	[618, 438, 11, 2, 669, 13, 18, 2]
9	370_to_372	374_to_376	0.092433	1.0	0.092433	Rec-Rec	[235, 0, 0, 27]
10	226	250_to_513	0.089469	0.184092	0.095802	('A', 'Pos', 'G', 'Neg')	[618, 406, 11, 2, 669, 11, 17, 3]
11	575	1218	0.084728	0.426624	0.135626	('A', 'A', 'G', 'C')	[181, 8, 4, 85, 4, 0, 0, 6, 59, 12, 8, 1291, 4, 0, 0, 8]
12	1217	1218	0.079553	0.794332	0.086432	('T', 'C', 'G', 'A')	[20, 4, 0, 13, 11, 2, 6, 1354, 143, 6, 2, 13, 2, 1, 0, 22]
13	1237	1234_to_1237	0.07839	0.912376	0.080484	('T', 'Neg', 'C', 'Pos')	[4, 0, 1292, 3, 3, 0, 7, 124]
14	736_to_1219	1223_to_1225	0.076266	0.164365	0.464003	Rec-Rec	[486, 430, 0, 193]
15	226	575	0.071722	0.125011	0.467635	('A', 'A', 'G', 'G')	[954, 20, 1143, 23, 11, 0, 11, 0, 38, 3, 650, 5, 4, 0, 23, 2]

Supplementary Table 2

Supplementary Table 2. The 15 associations with the highest wLD values detected in FHV RNA2 in Passage 6 (3sigma = 0.006272)

ID	NT1	NT2	wLD	wR2	wLDmax	correlation	Contingency table
1	250_to_513	736_to_1219	0.207513	0.709838	0.254205	Rec-Rec	[134, 12, 8, 103]
2	248_to_512	736_to_1219	0.20223	0.663083	0.246511	Rec-Rec	[134, 12, 13, 112]
3	1217	732_to_1216	0.169188	0.928924	0.174213	('T', 'Neg', 'G', 'Pos')	[3, 0, 150, 1, 1, 44, 0, 0]
4	575	248_to_512	0.166334	0.47638	0.197724	('A', 'Pos', 'G', 'Neg')	[14, 208, 2, 3, 158, 53, 1, 7]
5	575	250_to_513	0.163992	0.450745	0.197325	('A', 'Pos', 'G', 'Neg')	[14, 185, 2, 2, 158, 56, 1, 2]
6	1217	1218	0.159645	0.705782	0.174138	('T', 'C', 'G', 'A')	[7, 1, 0, 6, 5, 6, 1, 216, 79, 5, 1, 9, 1, 0, 1, 7]
7	1218	732_to_1216	0.159295	0.865189	0.164246	('A', 'Pos', 'C', 'Neg')	[3, 42, 3, 2, 0, 0, 151, 1]
8	575	736_to_1219	0.147404	0.378178	0.193736	('A', 'Pos', 'G', 'Neg')	[24, 241, 1, 5, 168, 74, 3, 2]
9	1218	250_to_513	0.134	0.474484	0.165746	('A', 'Pos', 'C', 'Neg')	[6, 39, 3, 1, 0, 4, 120, 16]
10	249_to_517	730_to_1229	0.133563	0.373929	0.137253	Rec-Rec	[143, 1, 58, 69]
11	1218	248_to_512	0.128188	0.421029	0.159601	('A', 'Pos', 'C', 'Neg')	[6, 39, 3, 1, 0, 1, 120, 21]
12	1218	734_to_1218	0.124059	0.80139	0.13453	('A', 'Pos', 'C', 'Neg')	[3, 29, 3, 1, 0, 0, 153, 2]
13	736_to_1219	1223_to_1225	0.123355	0.340403	0.36238	Rec-Rec	[48, 49, 0, 108]
14	1217	248_to_512	0.115831	0.546442	0.193212	('T', 'Neg', 'G', 'Pos')	[4, 1, 121, 13, 1, 26, 0, 2]
15	1224	734_to_1222	0.11202	0.432995	0.14202	('T', 'Neg', 'C', 'Pos')	[0, 0, 71, 3, 0, 1, 9, 16]

Supplementary Table 3

Supplementary Table 3. A226G and G575A are negatively associated with each other in FHV RNA2.

Passage	NT1	NT2	wLD	wR2	wLDmax	correlation	Contingency table
6	226	575	0.013454	0.014234	0.553561	('A', 'A', 'G', 'G')	[549, 16, 808, 13, 4, 0, 13, 0, 13, 1, 72, 1, 2, 0, 4, 0]
9	226	575	0.071722	0.125011	0.467635	('A', 'A', 'G', 'G')	[954, 20, 1143, 23, 11, 0, 11, 0, 38, 3, 650, 5, 4, 0, 23, 2]

Supplementary Table 4

Supplementary Table 4. Frequency of A226G in full-length RNA2 and D-RNA2 over passaging.

	A	G

Passage	All RNA2	In full-length RNA2	In D-RNA2	All RNA2	In full-length RNA2	In D-RNA2
1	0.96	NA	NA	0.03	NA	NA
2	0.93	NA	NA	0.06	NA	NA
3	0.97	NA	NA	0.02	NA	NA
4	0.83	0.73	0.99	0.16	0.25	0.01
5	0.92	0.79	0.97	0.07	0.19	0.01
6	0.94	0.75	0.96	0.06	0.22	0.02
7	0.69	0.45	0.97	0.29	0.53	0.02
8	0.83	0.54	0.95	0.16	0.44	0.03
9	0.74	0.49	0.96	0.25	0.49	0.03

NOTE: The frequency of SNV in wild-type RNA2 and defective RNA2 is calculated from the contingency table formed between SNV and the major recombination event 736^1219, which is not available in Passage 1, 2, and 3 due to the low frequency of the SNV and/or the recombination event 736^1219. NA, not available.

Supplementary Table 5

Supplementary Table 5. Frequency of G575A in full-length RNA2 and D-RNA2 over passaging.

	A			G		
Passage	All RNA2	In full-length RNA2	In D-RNA2	All RNA2	In full-length RNA2	In D-RNA2
1	0.02	NA	NA	0.97	NA	NA
2	0.02	NA	NA	0.97	NA	NA
3	0.02	NA	NA	0.97	NA	NA
4	0.11	0.04	0.81	0.89	0.95	0.17
5	0.26	0.06	0.74	0.73	0.93	0.24
6	0.38	0.12	0.75	0.61	0.86	0.23
7	0.22	0.03	0.77	0.77	0.95	0.17
8	0.47	0.07	0.86	0.52	0.90	0.11
9	0.32	0.04	0.81	0.68	0.95	0.16

NOTE: The frequency of SNV in wild-type RNA2 and defective RNA2 is calculated from the contingency table formed between SNV and the major recombination event 736^1219, which is not available in Passage 1, 2, and 3 due to the low frequency of the SNV and/or the recombination event 736^1219. NA, not available.

Supplementary Table 6

Supplementary Table 6. Common SNVs in each sample.

Locus	Ref	Alt	Alt_Frequency	Locus	Ref	Alt	Alt_Frequency	Locus	Ref	Alt	Alt_Frequency
**Sample No.1: Feb 1997**				**Sample No.2: Aug 2000**				**Sample No.3: Feb 2001**			
888	T	C	0.4	1033	G	A	0.57	832	C	A	0.51
915	A	G	0.3	909	G	A	0.44	1033	G	A	0.49
2442	A	G	0.26	1151	A	G	0.43	915	A	G	0.47
1995	C	T	0.23	915	A	G	0.4	1030	A	G	0.31
2466	G	A	0.23	960	T	C	0.29	1717	T	A	0.3
2436	G	A	0.16	834	A	G	0.28	1809	G	A	0.23
2497	C	T	0.16	852	A	G	0.27	960	T	C	0.19
1160	A	C	0.14	1809	G	A	0.26	909	A	G	0.16
1881	G	A	0.13	1030	A	G	0.22	888	T	C	0.12
2221	A	C	0.08	1717	T	A	0.17	1995	C	T	0.11
**Sample No.4: Oct 2001**				**Sample No.5: Jun 2002**							
1717	T	A	0.54	2390	G	A	0.42				
1030	A	G	0.39	1263	A	C	0.41				
1033	G	A	0.39	1977	C	T	0.41				
1155	A	C	0.36	889	A	G	0.37				
832	C	A	0.33	2467	T	C	0.34				
2412	A	G	0.33	1432	G	T	0.31				
2517	C	A	0.2	1479	A	G	0.29				
2534	T	C	0.19	978	G	A	0.29				
1263	A	C	0.18	1356	C	T	0.28				
1809	G	A	0.17	1995	C	T	0.26				

NOTE: NGS data from each sample is mapped to the HXB2-indexed consensus genome of that sample.

Supplementary Table 7

Supplementary Table 7. Associations involving the RxEPS insertion in the p6^{Gag} detected by CoVaMa in Sample 4. (3sigma = 0.027592)

NT1	NT2	wLD	wR2	wLDmax	correlation	Contingency table

2146	2157_AGACCAGAGCCATCA_2158	0.046153	0.451311	0.062868	('T', 'Pos', 'C', 'Neg')	[3, 0, 220, 680, 6, 0, 11873, 380]
2146	2157_AGACTAGAGCCATCA_2158	0.028763	0.581251	0.030682	('T', 'Pos', 'C', 'Neg')	[3, 0, 220, 379, 6, 0, 11873, 24]
2211	2157_AGACCAGAGCCATCA_2158	0.00815	0.007943	0.05387	('A', 'Pos', 'G', 'Neg')	[1364, 175, 0, 0, 7310, 424, 1, 0]
2201	2157_AGACCAGAGCCATCA_2158	0.005183	0.006018	0.005183	('T', 'Neg', 'C', 'Pos')	[0, 0, 722, 0, 2, 0, 8702, 742]
2198	2157_AGACCAGAGCCATCA_2158	0.004705	0.006266	0.053864	('T', 'Pos', 'C', 'Neg')	[1, 0, 529, 94, 1, 0, 9481, 655]
2200	2157_AGACCAGAGCCATCA_2158	0.0045	0.004878	0.004792	('A', 'Pos', 'G', 'Neg')	[8836, 746, 0, 0, 670, 3, 1, 1]
2100	2157_AGACCAGAGCCATCA_2158	0.004057	0.004686	0.004057	('T', 'Neg', 'C', 'Pos')	[0, 0, 123, 0, 0, 0, 2502, 278]
2197	2157_AGACTAGAGCCATCA_2158	0.003539	0.01154	0.027437	('A', 'Neg', 'G', 'Pos')	[9742, 249, 3, 0, 375, 49, 1, 0]
2214	2157_AGACCAGAGCCATCA_2158	0.003504	0.010557	0.018263	('A', 'Pos', 'G', 'Neg')	[133, 43, 1, 0, 8291, 542, 1, 0]
2211	2157_AGACTAGAGCCATCA_2158	0.002959	0.002832	0.003747	('A', 'Neg', 'G', 'Pos')	[1364, 7, 0, 0, 7310, 209, 1, 0]
2206	2157_AGACCAGAGCCATCA_2158	0.002584	0.002428	0.003092	('T', 'Pos', 'C', 'Neg')	[3, 0, 8728, 662, 1, 0, 444, 5]
2147	2158_AGACCAGAGCCATCA_2158	0.002247	0.002515	0.002247	('T', 'Neg', 'C', 'Pos')	[3, 0, 367, 0, 8, 0, 11724, 1060]
2169	2157_AGACCAGAGCCATCA_2158	0.002185	0.003019	0.080219	('A', 'Neg', 'G', 'Pos')	[11406, 989, 3, 0, 222, 50, 3, 1]
2201	2157_AGACTAGAGCCATCA_2158	0.002117	0.002355	0.002117	('T', 'Neg', 'C', 'Pos')	[0, 0, 722, 0, 2, 0, 8702, 276]
2200	2157_AGACTAGAGCCATCA_2158	0.001959	0.002165	0.001959	('A', 'Pos', 'G', 'Neg')	[8836, 280, 0, 0, 670, 0, 1, 0]

Supplementary Table 8

Supplementary Table 8. Associations involving the RxEPS insertion in the $p6^{Gag}$ detected by CoVaMa in Sample 1. (3sigma = 0.076140)

NT1	NT2	wLD	wR2	wLDmax	correlation	Contingency table
2096	2157_AGACCAGAGCCATCA_2158	0.129189	0.312063	0.151065	('A', 'Pos', 'G', 'Neg')	[678, 1401, 0, 0, 871, 66, 0, 1]
2081	2157_AGACCAGAGCCATCA_2158	0.024842	0.038874	0.03672	('T', 'Pos', 'C', 'Neg')	[0, 0, 26, 124, 0, 0, 1147, 892]
2221	2157_AGACCAGAGCCATCA_2158	0.018924	0.020563	0.026611	('A', 'Pos', 'C', 'Neg')	[4448, 1873, 4, 1, 16, 3, 625, 54]
2166	2157_AGACCAGAGCCATCA_2158	0.017251	0.02066	0.349795	('A', 'Neg', 'G', 'Pos')	[7116, 3936, 2, 3, 283, 496, 0, 0]
2097	2157_AGACCAGAGCCATCA_2158	0.015503	0.028963	0.016443	('A', 'Pos', 'G', 'Neg')	[1562, 1521, 0, 0, 107, 3, 0, 0]
2155	2157_AGACCAGAGCCATCA_2158	0.00944	0.015457	0.00944	('A', 'Neg', 'T', 'Pos')	[301, 0, 7141, 4435, 0, 0, 30, 0]
2130	2157_AGACCAGAGCCATCA_2158	0.009365	0.019293	0.00949	('T', 'Neg', 'C', 'Pos')	[0, 0, 148, 1, 0, 0, 3775, 4113]
2136	2157_AGACCAGAGCCATCA_2158	0.009121	0.01863	0.009236	('T', 'Neg', 'C', 'Pos')	[0, 1, 158, 1, 0, 1, 4144, 4432]
2148	2157_AGACCAGAGCCATCA_2158	0.006533	0.008644	0.008044	('A', 'Pos', 'C', 'Neg')	[7213, 4410, 1, 2, 20, 5, 239, 18]
2190	2157_AGACCAGAGCCATCA_2158	0.006102	0.008152	0.007077	('A', 'Neg', 'G', 'Pos')	[203, 10, 0, 2, 6558, 3487, 0, 1]
2211	2157_AGACCAGAGCCATCA_2158	0.002351	0.001924	0.009928	('A', 'Pos', 'G', 'Neg')	[62, 53, 0, 0, 5718, 2349, 1, 0]
2100	2157_AGACCAGAGCCATCA_2158	0.001952	0.000289	0.029112	('T', 'Pos', 'C', 'Neg')	[0, 0, 94, 100, 0, 1, 1703, 1563]

Supplementary Table 9

Supplementary Table 9.
Frequency of SNVs in reads with and without 'RLEPS' insertion in longitudinal samples.

	Frequency of A2096G (K436R) in the NC/P1 cleavage site (%)			Frequency of C2146T (P453S) in the p1/p6 cleavage site (%)		
Sample No.	In all reads	In reads without insertion	In reads with insertion	In all reads	In reads without insertion	In reads with insertion
1	26	NA	NA	0	NA	NA
2	9	14.01	6.56	2	0.97	27.95

3	10	14.26	5.41	7	1.69	17.42
4	1	NA	NA	13	1.82	94.04
5	0	NA	NA	23	4.92	99.18

**Frequency of SNVs in reads with and without 'RPEPS' insertion in longitudinal samples**						
	Frequency of A2096G (K436R) in the NC/P1 cleavage site (%)			Frequency of C2146T (P453S) in the p1/p6 cleavage site (%)		
Sample	In all reads	In reads without insertion	In reads with insertion	In all reads	In reads without insertion	In reads with insertion
1	26	56.23	4.50	0	NA	NA
2	9	14.01	1.34	2	0.97	51.61
3	10	14.26	1.03	7	1.69	73.15
4	1	NA	NA	13	1.82	64.15
5	0	NA	NA	23	4.92	68.84

NOTE: The frequency of SNVs in reads with and without 'RxEPS' insertion is calculated from the contingency table, which is not available when the frequency of the SNV and/or insertion event is lower than the detection level. NA, not available.

Supplementary Table 10.

Supplementary Table 10. Contingency table for the association between 2096 and 2146 (sample 3).

2096 \ 2146	T (P453S)	C
A	439	3302
G (K436R)	14	590

Supplementary Table 11

Supplementary Table 11. Contingency table for the association between C2081T and 'RPEPS' insertion (sample 1).

2081 \ 'RPEPS' insertion	Negative	Positive
T (A431V)	26	124
C	1147	892

Supplementary Figure 1. A185T in the capsid protein locates at the quasi-three-fold and five-fold symmetry axes of the Flock House virus particle. This capsid structure depicts the location of G575A (Alanine to Threonine substitution at amino acid 185) at quasi-three-fold and five-fold symmetry axes of the Flock House virus particle. The structure is modified based on 4FTB.

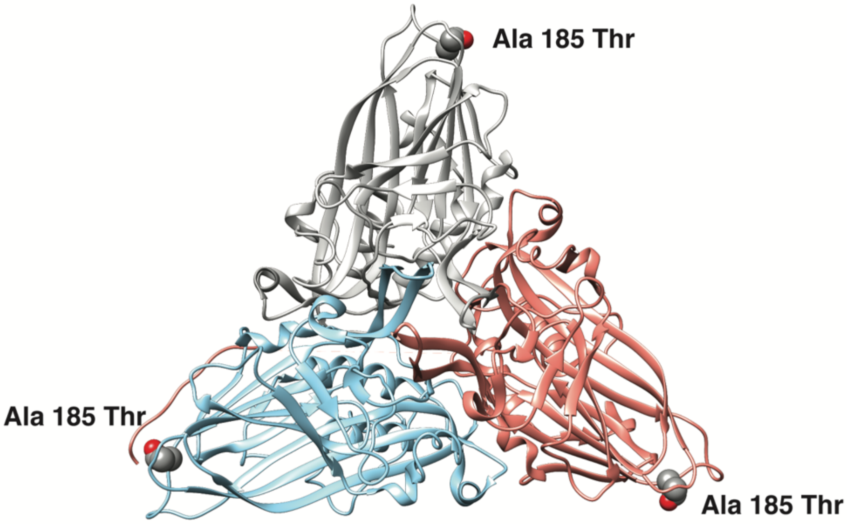

Supplementary Figure 2. Associations between recombination events and SNVs in FHV RNA2 in Passage 6. The 15 associations with the highest wLD values in Passage 6 revealed by CoVaMa are labeled from 1 to 15. (A) The wLD values of the 15 associations are plotted from high to low. The associations between major recombination events are labeled in the plot. The three-sigma threshold is shown by the red dashed line. (B) This schematic diagram shows the distribution of the 15 associations on FHV RNA2. Recombination events and InDels are plotted using blocks and SNVs are plotted using dots.

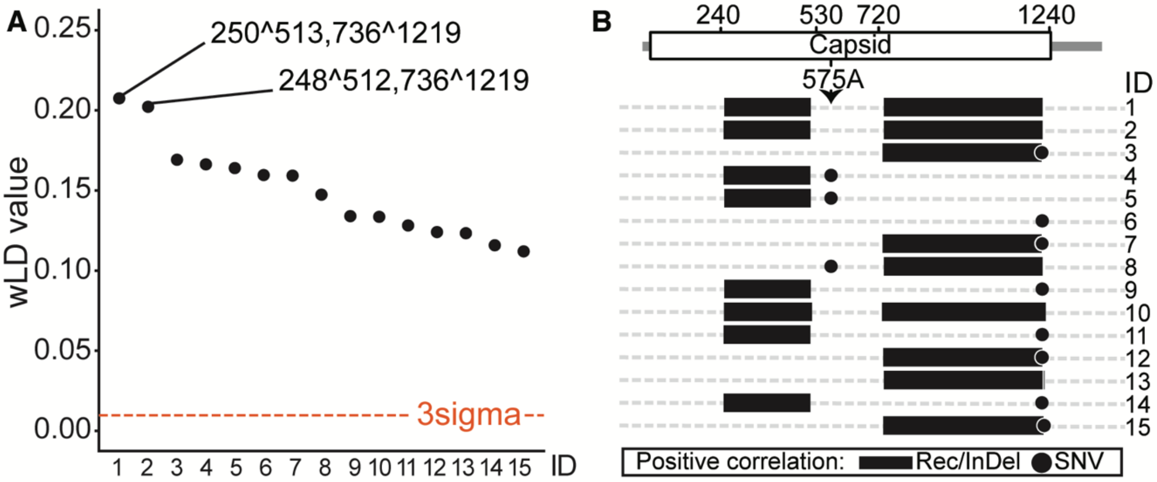

Supplement: gkab1259_Supplemental_Files [file gkab1259_supplemental_files.zip › Wang_2021_SupplementaryData_part1_NAR.pdf]
